# Sex differences and age-related changes in the mandibular alveolar bone mineral density using a computer-aided measurement system for intraoral radiography

**DOI:** 10.1038/s41598-024-57805-5

**Published:** 2024-03-28

**Authors:** Ryutaro Ono, Akitoshi Katsumata, Yumi Fujikawa, Emi Takahira, Toshiro Yamamoto, Narisato Kanamura

**Affiliations:** 1 Department of Dental Medicine, Graduate School of Medicine, Kyoto Prefectural University of Medicine, 465 Kajii-cho, Kawaramachi-Hirokoji, Kamigyo-ku, Kyoto, 602-8566 Japan; 2 Department of Oral and Maxillofacial Surgery, North Medical Center, Kyoto Prefectural University of Medicine, Kyoto, Japan; 3 Department of Oral Radiology, Asahi University School of Dentistry, Gifu, Japan

**Keywords:** Dental radiology, Software

## Abstract

This study aimed to conduct a cross-sectional data analysis of the alveolar bone mineral density (al-BMD) in 225 patients of various ages and different sexes. The al-BMD value in the mandibular incisor region was calculated using a computer-aided measurement system (DentalSCOPE) for intraoral radiography. All participants with intact teeth (101 males and 124 females; age range, 25–89 years) were divided into three age-segregated groups (25–49, 50–74, and > 75 years). Statistical differences were evaluated using the Mann–Whitney U or Kruskal–Wallis test. Males exhibited significantly greater al-BMD than females (p < 0.001). The highest means were observed in the 25–49 age group, regardless of sex (1007.90 mg/cm^2^ in males, 910.90 mg/cm^2^ in females). A 9.8% decrease in al-BMD was observed with the increase in age in males (25–49 to 50–74 years; p = 0.004); however, no further changes were seen thereafter. In females, a decreasing trend was seen throughout the lifespan, with values reaching up to 76.0% of the initial peak value (p < 0.001). Similar to other skeletal sites, the alveolar bone exhibits sex differences and undergoes a reduction in BMD via the normal aging process.

## Introduction

**Figure 1:**
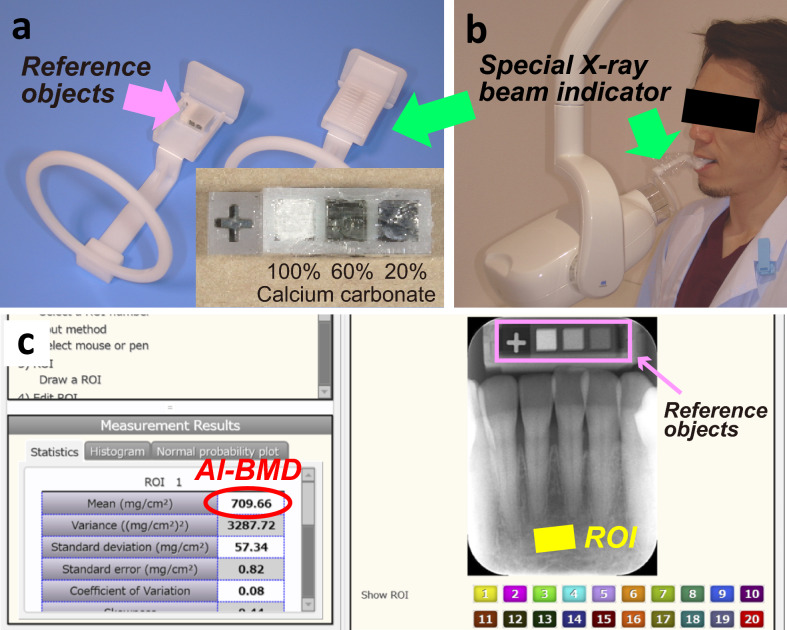
A computer-aided measurement system DentalSCOPE. (**a**,**b**) The DentalSCOPE device consists of an X-ray beam indicator built-in special reference objects which calibrated to a specific bone mineral density (BMD). The right lower panel shows an enlarged view of the reference phantoms (500, 1000, and 1500 mg/cm^2^, respectively). (**c**) Measurements and calculations of the alveolar-BMD (al-BMD) using the DentalSCOPE software (Details methodology are outlined in the Methods section). The region of interest (ROI) is represented as a yellow-colored rectangular area (12–14 mm^2^) 3 mm away from the central incisor root apex (in the vertical direction).

Bone mineral density (BMD) is one of the major determinants of bone strength. Therefore, measurements of BMD can be widely used for diagnosing osteoporosis, predicting fracture risk, and monitoring the bone changes associated with treatment^1,2^. In dentistry, visual classifications of the alveolar trabecular pattern seen on intraoral radiographs have been thought to be a helpful indicator of skeletal BMD^3,4^. However, the accuracy of this qualitative diagnostic approach is mainly dependent on the radiographic interpretation skills of dental professionals. Recently, a computer-aided measurement system DentalSCOPE (Fig. 1) based on conventional micro-densitometry techniques, has been developed. This device facilitates the calculation of the alveolar-BMD (al-BMD) value in an arbitrary region and may have a critical role in quantitative imaging techniques^5,6^. Considering the limited number of publications on this topic, the present study aimed to conduct a cross-sectional data analysis of the al-BMD in 225 participants in order to evaluate the sex differences and age-related changes in the mandibular alveolar bone using a computer-aided measurement system for intraoral radiography.

## Results

### Demographic characteristics and menopausal status

The study subjects consisted of 101 males (44.9%) and 124 females (55.1%) with an age range of 25–89 years (mean ± standard deviation, 61.4 ± 15.2 years). As shown in Supplementary Fig. S1, the mean and median values were not equal because a higher number of middle-aged to elderly patients compared to younger patients were examined in this study. Supplementary Table S1 presents the number and proportion of study participants; just over half (53.5% males and 55.7% females) belonged to the 50–74 age group. No significant difference for mean age was found between two the sexes. The majority (63/69, 91.3%) of women in the 50–74 age group and all those aged > 75 years of age (22/22) had reached menopause compared to only 3 of 33 (9.1%) women in the 25–49 age group (see Supplementary Fig. S2 online).

### Comparison of al-BMD values based on sex and age

**Figure 2:**
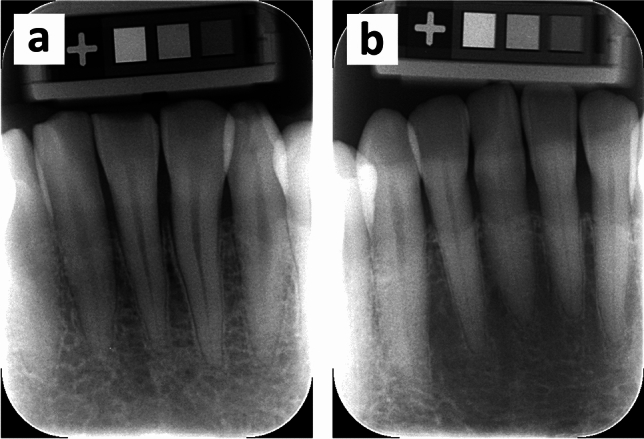
Two representative radiographic images demonstrating the highest (**a**: 1259.26 mg/cm^2^, male, 28 years) and lowest (**b**: 495.24 mg/cm^2^, female, 78 years) al-BMD values in the analysis. Dense trabeculation (**a**) and sparse trabeculation pattern (**b**) are predominant. Marginal bone loss is commonly less than two-thirds of the root length.

Two representative examples of intraoral radiography demonstrating the highest and lowest al-BMD values are shown in Fig. 2 (a: 1259.26 mg/cm^2^, male 28 years; b: 495.24 mg/cm^2^, female 78 years). As seen in Fig. 2a, well-mineralized trabeculae, and small intertrabecular spaces were predominantly observed within the periapical region of the incisors. Conversely, the trabeculation pattern in Fig. 2b was sparse and irregular.

**Figure 3:**
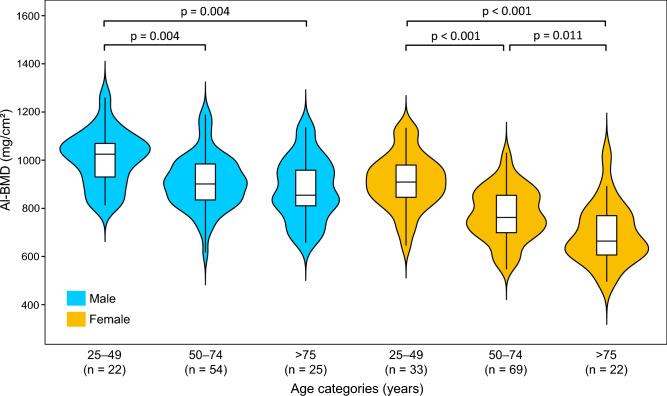
Box and violin plots of the al-BMD stratified by sex and age (25–49, 50–74, and > 75 years). The horizontal line inside the box represents the median of all values, and the box ends show the 25th and 75th percentiles. The whiskers mark the range within 1.5 times the interquartile distance. The Kruskal–Wallis test, followed by the pairwise Wilcox test, was performed.

Table 1 shows the summary of the al-BMD measurements. The average al-BMD values were significantly (p < 0.001) greater among males (923.30 ± 124.81 mg/cm^2^) than females (795.99 ± 135.30 mg/cm^2^; see Supplementary Fig. S3 online). A similar trend was evident in each age group (p = 0.004 in the 25–49 group; p < 0.001 in the 50–74 and > 75 groups). The highest mean values were observed in young to middle-aged adults (25–49 years), regardless of sex (1007.90 ± 113.40 mg/cm^2^ in males and 910.90 ± 115.16 mg/cm^2^ in females; Fig. 3). The al-BMD in males aged 50–74 years had significantly decreased (p = 0.004) by ~ 9.8% from that observed in those aged 25–49; no further changes were detected thereafter. In females, the al-BMD showed a decreasing trend throughout the lifespan until it reached a value of 76.0% that of the initial peak value (25–49 vs. 50–74 years, p < 0.001; 50–74 vs. > 75 years, p = 0.011).

**Table 1:** Summary of the alveolar bone mineral density (al-BMD) measurements.

Age categories^1^	N (%)	Mean	S.D	P-value	Post-hoc
Male					
(a) 25–49 years	22 (21.8%)	1007.90	113.40	0.001	a vs. b
(b) 50–74 years	54 (53.5%)	908.93	112.49		a vs. c
(c) > 75 years	25 (24.8%)	879.90	129.29		
Female					
(a) 25–49 years	33 (26.6%)	910.90	115.16	< 0.001	a vs. b
(b) 50–74 years	69 (55.7%)	774.00	108.66		a vs. c
(c) > 75 years	22 (17.7%)	692.62	123.49		b vs. c

^1^Kruskal–Wallis test followed by pairwise Wilcox test as post hoc analysis.

Vs. indicates significant differences between the two groups.

*S.D*., standard deviation.

## Discussion

In the present study, the al-BMD values in 225 patients of various ages (range, 25–89 years) and different sexes were calculated using a recently developed computer-aided measurement system DentalSCOPE. Before and during the course of dental treatment, a cone-beam computed tomography (CBCT) is increasingly used in the radiographic evaluation of jawbone morphology. However, due to various problems, such equipment is not always accessible at all facilities. Therefore, fundamental information on the al-BMD is in demand and may serve the basis towards further developments of new alternative/complementary diagnostic concept covering wide range of CBCT-guided procedures, such as predicting implant stability^7^, position decision of the orthodontic miniscrew^8^, and early detection of osteonecrosis incidence^9^.

Males exhibited significantly higher al-BMD values than females (Table 1 and see Supplementary Fig. S3 online), which is consistent with the results of a previous study conducted in the Chinese population (55–64 age group)^10^. However, Kazazić et al.^11^ reported no significant influence of sex on al-BMD in the maxillary anterior teeth. In this study, the mandible was considered appropriate for the site of measurement to evaluate the skeletal status^12^. The alveolar process consists of a thin outer cortex and an adjacent cancellous bone with many trabeculae. Considering the significant correlation coefficient between CT value of cancellous bone and BMD in mandibular alveolar region^13^, this discrepancy in the result was probably attributable to the difference in the distribution of trabecular components in the two jaws.

Optimal bone metabolism is a dynamic process controlled by hormonal, nutritional, and mechanical harmony^14^. The annual increase in BMD is most significant in the late adolescent stage, following which it reaches the peak value within the first two decades of human life^15,16^. A recent study presented the computer-assisted measurements of radiographical al-BMD in the early 20 s people, whose values closely resembled our results^5^. Nonetheless, there is a lack of consensus on how advancing age can impact the conditions of the alveolar bone. In this study, we determined the al-BMD in patients aged 25–89. A decrease in al-BMD was noted during the middle adulthood stage (Fig. 2). After menopause, women experienced a notable age-related decrease in al-BMD similar to that observed in other skeletal sites^17^, which can be explained by the increase in bone resorption under estrogen-deficient conditions^18^. Alveolar osteoblasts from elderly women, even at the in vitro level, showed a reduced proliferative capacity and bone formation functions^19^. There is also an age-progressive increase in the number of mouse bone marrow cells which capable of forming osteoclasts^20^. Conversely, a clear downward trend was not seen in elderly males, which suggested that the density of mandibular alveolar bone would remain virtually constant throughout the rest of lifespan. A detailed link between the aging process and al-BMD changes are not well elucidated in the current study.

Nowadays, more people visit the dental office for regular check-ups, and intraoral radiographs are routinely taken to obtain diagnostic information at low doses of radiation exposure. Jonasson et al.^3,4^ validated a three-scale visualized classification method for the mandibular trabecular patterns to predict osteoporosis; a dense trabeculation in the alveolar process was a strong indicator of high skeletal BMD, whereas a sparse trabecular pattern may predict osteopenia. However, this type of qualitative assessment remains challenging for nondental radiologists. DentalSCOPE may overcome these difficulties and allow general dentists to conduct quantitative measurements of the al-BMD in the normal clinical setting.

This study has some limitations. First, because of the cross-sectional design, there may have been unmeasured confounding factors, which could influence the observed outcomes. Therefore, prospective data collection from the same individuals might prove beneficial for detecting any changes that occur in the alveolar bone over an extended period. Second, the patients in this study were cognitively intact and maintained relatively good oral health. Thus, it might be challenging to identify the position of the original alveolar ridge in such patients, especially when targeting those with few remaining anterior teeth or edentulous. Third, the BMD was not measured in this population sample, therefore making it difficult to directly evaluate the relationship between a low al-BMD and the prevalence of osteoporosis. In one study, Takaishi et al.^21^ used a different computed radiogrammetry technique to demonstrate the correlation between the al-BMD in the mandible and the incidence of osteoporotic spinal fracture in postmenopausal women. Therefore, additional studies confirming the reliability of the al-BMD measurement using DentalSCOPE for the early detection of undiagnosed osteoporosis are warranted.

## Methods

### Study design and participants

This cross-sectional survey was conducted at the North Medical Center Kyoto Prefectural University of Medicine, from March 2022 to January 2023. A total of 261 patients (male, 112; females, 149) at the Department of Oral and Maxillofacial Surgery volunteered to be included in the study. Data from radiographic examinations, questionnaires (see Supplementary Note S1 online), and medical records were collected and used for the study. Written informed consent for the publication of any potentially identifiable data or images included in this study was obtained from all subjects and/or their legal guardian(s) prior to conducting any research activity. The study was approved by the Ethical Committee of the Kyoto Prefectural University of Medicine (approved number: ERB-C-2265). All experiments were performed in accordance with the Declaration of Helsinki.

A detailed flow diagram of the inclusion and exclusion process is illustrated in Supplementary Fig. S4. The exclusion criteria in this study included the following: provision of inconsistent responses in the questionnaire (N = 2); patients who underwent radiotherapy for the jaw (N = 3); those who have been diagnosed with osteoporosis and received medications to treat (N = 13); and those who presented with periodontal bone loss (> two-thirds of the root length; N = 4), apical radiolucency (> 3 mm in diameter; N = 4), missing teeth in the mandibular anterior region (N = 7), and extensive mandibular torus in the intraoral radiographs (N = 3). Based on these criteria, 225 patients were enrolled and divided into three mutually exclusive groups: 25–49, 50–74, and > 75 years old. The age categorization was performed according to the previous literatures on bone health indices^17,22,23^.

### Dental radiographic image acquisition and measurements of the alveolar BMD value

Digital periapical radiographs of the mandibular incisor region were acquired for research purposes using intraoral X-ray equipment (maxiX Type2^®^, J. MORITA MFG. Corp., Kyoto, Japan) with an imaging plate system (Digora Optime^®^, Soredex, Orion Corp., Helsinki, Finland) under the following conditions: tube voltage, 60 kV; electrical current, 7 mA, and exposure time, 0.13 s. The DentalSCOPE, version 1.0.2 (Media Corp., Tokyo, Japan, https://md-scope.com/products/dental/index.html [provided in Japanese only]) system was used to evaluate the alveolar-BMD (al-BMD). Under certification by the national health institute in the authors’ nation, an X-ray beam indicator (Fig. 1a,b) was designed to built-in special reference objects which comprised 20% (calibrated to 500 mg/cm^2^), 60% (1000 mg/cm^2^), and 100% (1500 mg/cm^2^) calcium carbonate. The mg/cm^2^ is a convenient unit to express the value of al-BMD determined from two-dimensional X-ray images. After inputting IP-based digital X-ray images, the computer-aided detection software automatically created a calibration line based on the image densities of the reference phantoms and converted each image density of the arbitrary region into a BMD value. A 12–14 mm^2^ rectangular area, 3 mm away (in the vertical direction) from the central incisor root apex, was selected as the region of interest (Fig. 1c) due to lower incidence of anatomical variance, such as torus mandibularis^24^ or idiopathic osteosclerosis^25^ for analysis. Three measurements were taken for each subject blindly every 1 week by single well-trained examiner, and the median value was recorded as the representative data.

### Questionnaire survey

The questionnaire used in this study (see Supplementary Note S1 online) included two items about the individual’s basic demographic information: Q1. What is your sex (male/ female), Q2. What is your age (in years)? Two questions regarding osteoporosis diagnosis and treatment were also provided: Q3. Have you ever been diagnosed with osteoporosis by a physician? (yes/no), Q4. For those who answered “yes” to the previous question; Are you currently or have you ever taken any of the following medications for osteoporosis, such as bisphosphonates, denosumab, teriparatide, estrogens, raloxifene, and calcitonin? Additionally, women answered the following question: Q5. What is your menopausal status (premenopausal/ postmenopausal)?

### Statistical analysis

First, based on the Shapiro–Wilk test, each age- and sex-stratified group showed a significant departure from normality distribution of the al-BMD (p = 0.006–0.032). The Levene’s test for homogeneity of variance was statistically insignificant (F = 0.851, p = 0.403), indicating the equal variance. The al-BMD values were therefore compared using the nonparametric Mann–Whitney U test or Kruskal–Wallis test, followed by the pairwise Wilcox test as post-hoc analysis. All statistical analyses were performed using the R software package, version 4.1.0 (R Foundation for Statistical Computing, Vienna, Austria). A significance level of < 0.05 was considered significant.

### Approval for human experiments

Informed consent was obtained from all subjects and/or their legal guardian(s) for study participation.

### Supplementary Information

Supplementary Note S1.

Supplementary Figure S1.

Supplementary Figure S2.

Supplementary Figure S3.

Supplementary Figure S4.

Supplementary Table S1.

## Data Availability

The data that support the findings of this study are available on request from the corresponding author.

## References

[1] Kanis JA (2002). Diagnosis of osteoporosis and assessment of fracture risk. Lancet.

[2] Buckley L (2017). 2017 American College of Rheumatology guideline for the prevention and treatment of glucocorticoid-induced osteoporosis. Arthritis Rheumatol..

[3] Jonasson G, Bankvall G, Kiliaridis S (2001). Estimation of skeletal bone mineral density by means of the trabecular pattern of the alveolar bone, its interdental thickness, and the bone mass of the mandible. Oral Surg. Oral Med. Oral Pathol. Oral Radiol. Endod..

[4] Jonasson G, Skoglund I, Rythén M (2018). The rise and fall of the alveolar process: Dependency of teeth and metabolic aspects. Arch. Oral Biol..

[5] Oohashi M (2020). Computer-assisted measurement of radiographical alveolar bone density using intraoral radiographs: Preliminary study on comparison between men and women in young adults. J. Oral Maxillofac. Radiol..

[6] Mizuhashi R (2021). Quantitative periapical radiography using computer-assisted measurement for intraoral projections. J. Oral Maxillofac. Radiol..

[7] Noaman AT, Bede SY (2022). The effect of bone density measured by cone beam computed tomography and implant dimensions on the stability of dental implants. J. Craniofac Surg..

[8] Ronsivalle V (2023). Accuracy of digital workflow for placing orthodontic miniscrews using generic and licensed open systems. A 3d imaging analysis of non-native .stl files for guided protocols. BMC Oral Health.

[9] Ogura I (2021). CBCT imaging and histopathological characteristics of osteoradionecrosis and medication-related osteonecrosis of the jaw. Imaging Sci. Dent..

[10] Wen N (2000). 2000 Mineral density of alveolar bone after tooth extraction in the male and female. J. Pract. Stomatol..

[11] Kazazi L (2019). Influence of age and gender on alveolar bone density in patients with fixed prosthetics. Stomatol. Rev..

[12] Pluskiewicz W, Tarnawska B, Drozdzowska B (2000). Mandibular bone mineral density measured using dual-energy X-ray absorptiometry: Relationship to hip bone mineral density and quantitative ultrasound at calcaneus and hand phalanges. Br. J. Radiol..

[13] Katsumata A, Kohinata K, Esaki Y, Kawai M (2022). Variance of radiographical alveolar bone mineral density by the anatomical morphology of mandibular bone. Heliyon.

[14] Borer KT (2005). Physical activity in the prevention and amelioration of osteoporosis in women: Interaction of mechanical, hormonal and dietary factors. Sports Med..

[15] Kröger H, Kotaniemi A, Kröger L, Alhava E (1993). Development of bone mass and bone density of the spine and femoral neck–a prospective study of 65 children and adolescents. Bone Miner..

[16] Boot AM (2010). Peak bone mineral density, lean body mass and fractures. Bone.

[17] Warming L, Hassager C, Christiansen C (2002). Changes in bone mineral density with age in men and women: A longitudinal study. Osteoporos. Int..

[18] Raisz LG (2005). Pathogenesis of osteoporosis: Concepts, conflicts, and prospects. J. Clin. Invest..

[19] Jiang SY, Shu R, Xie YF, Zhang SY (2010). Age-related changes in biological characteristics of human alveolar osteoblasts. Cell Prolif..

[20] Kahn A, Gibbons R, Perkins S, Gazit D (1995). Age-related bone loss. A hypothesis and initial assessment in mice. Clin. Orthop. Relat. Res..

[21] Takaishi Y (2013). Assessment of alveolar bone mineral density as a predictor of lumbar fracture probability. Adv. Ther..

[22] Berger C (2008). Change in bone mineral density as a function of age in women and men and association with the use of antiresorptive agents. CMAJ.

[23] Liu M (2014). The effect of age on the changes in bone mineral density and osteoporosis detection rates in Han Chinese men over the age of 50. Aging Male.

[24] Hiremath VK, Husein A, Mishra N (2011). Prevalence of torus palatinus and torus mandibularis among Malay population. J. Int. Soc. Prev. Community Dent..

[25] Miloglu O, Yalcin E, Buyukkurt MC, Acemoglu H (2009). The frequency and characteristics of idiopathic osteosclerosis and condensing osteitis lesions in a Turkish patient population. Med. Oral Patol. Oral Cir. Bucal.

