# Supplementary material for: Sex differences and age-related changes in the mandibular alveolar bone mineral density using a computer-aided measurement system for intraoral radiography
Source: Sci Rep. 2024 Mar 28;14:7386. doi: 10.1038/s41598-024-57805-5 (PMC10979020; doi:10.1038/s41598-024-57805-5)
Supplement: Supplementary file 1 — Supplementary Note S1. [file 41598_2024_57805_MOESM1_ESM.docx]

**Supplementary Note S1**

Dear Respondents:

This questionnaire survey collects information to better understand the patients’ overall medical conditions. Kindly respond to all questions by filling in the blank spaces provided or putting a tick [**√**]. Your answers are only used for the research purpose and will be kept confidential. It takes a total of 5–10 minutes.

Your Name (optional):

Patient’s ID Number:

Date of Response: / /

**Basic demographic information**

1. What is your sex?

Male 　　　 Female

2. What is your age?

in years

**Osteoporosis diagnosis**

3. Have you ever been diagnosed with osteoporosis by a physician?

Yes 　　　 No

4. (For those who answered “Yes” to the previous question) Are you currently or have you ever taken any of the following medications for the treatment?

Yes 　　　 No

　↓

Bisphosphonates

Denosumab

Teriparatide

Estrogens

Raloxifene

Calcitonin

**Menopausal status**

5. (A question for women) What is your menopausal status?

Premenopausal (Regular menstrual

cycles in 22–35 days range)

Postmenopausal (No periods for at

least 12 months)

Thank you for taking the time to complete our questionnaire survey. Please return the document to the staff members.
