# Supplementary figures and images for: Sex differences and age-related changes in the mandibular alveolar bone mineral density using a computer-aided measurement system for intraoral radiography
Source: Sci Rep. 2024 Mar 28;14:7386. doi: 10.1038/s41598-024-57805-5 (PMC10979020; doi:10.1038/s41598-024-57805-5)

## Supplementary Figure S1

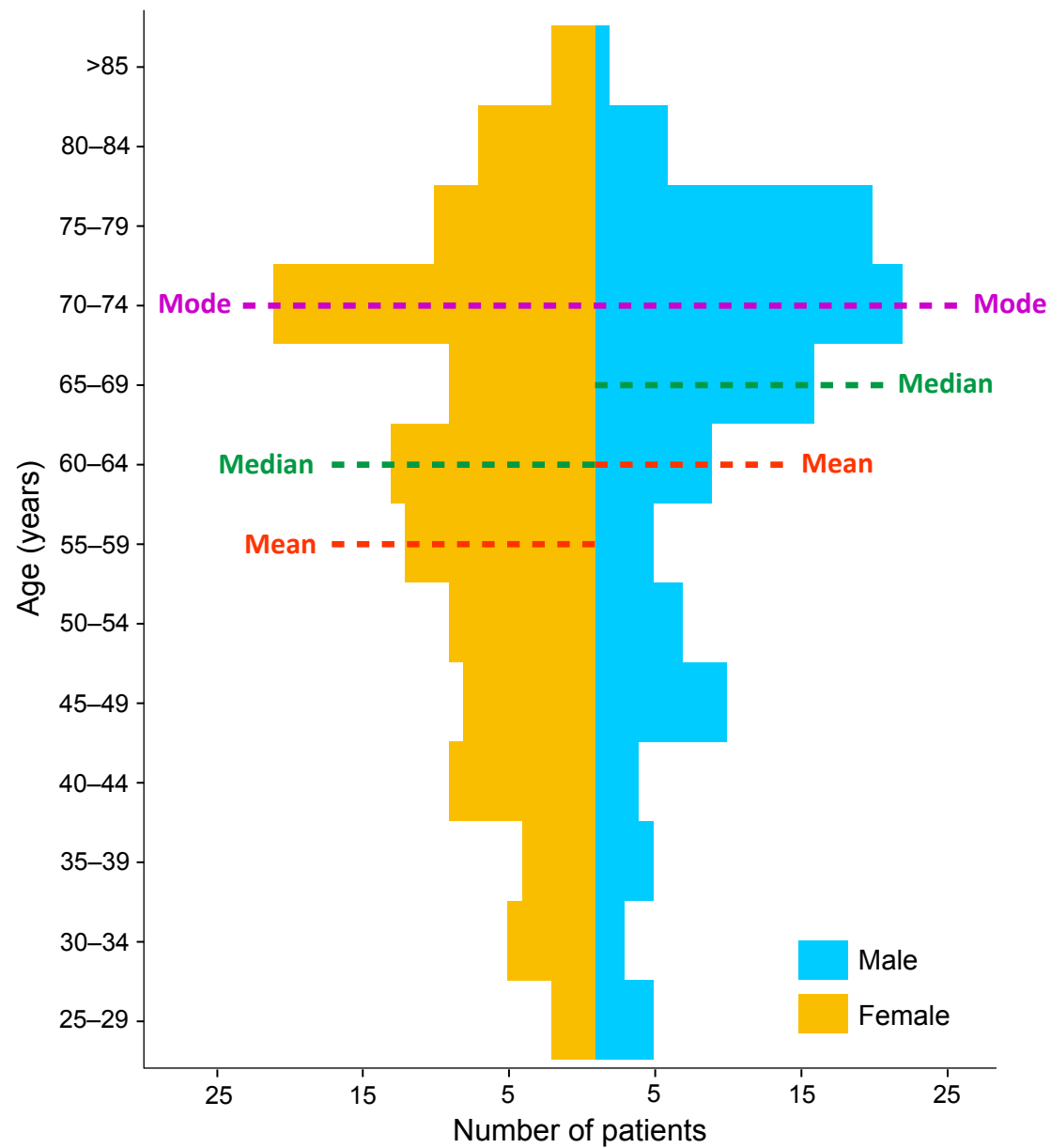

Supplement: Supplementary file 2 — Supplementary Figure S1. [file 41598_2024_57805_MOESM2_ESM.pdf]

## Supplementary Figure S2

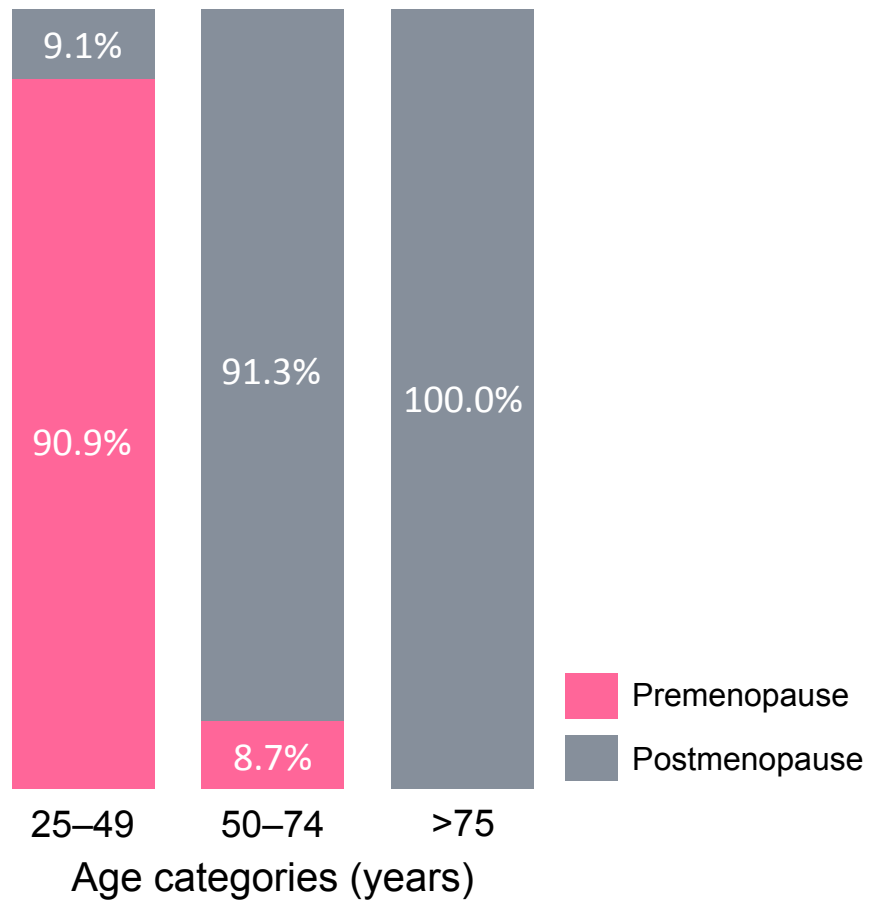

Supplement: Supplementary file 3 — Supplementary Figure S2. [file 41598_2024_57805_MOESM3_ESM.pdf]

## Supplementary Figure S3

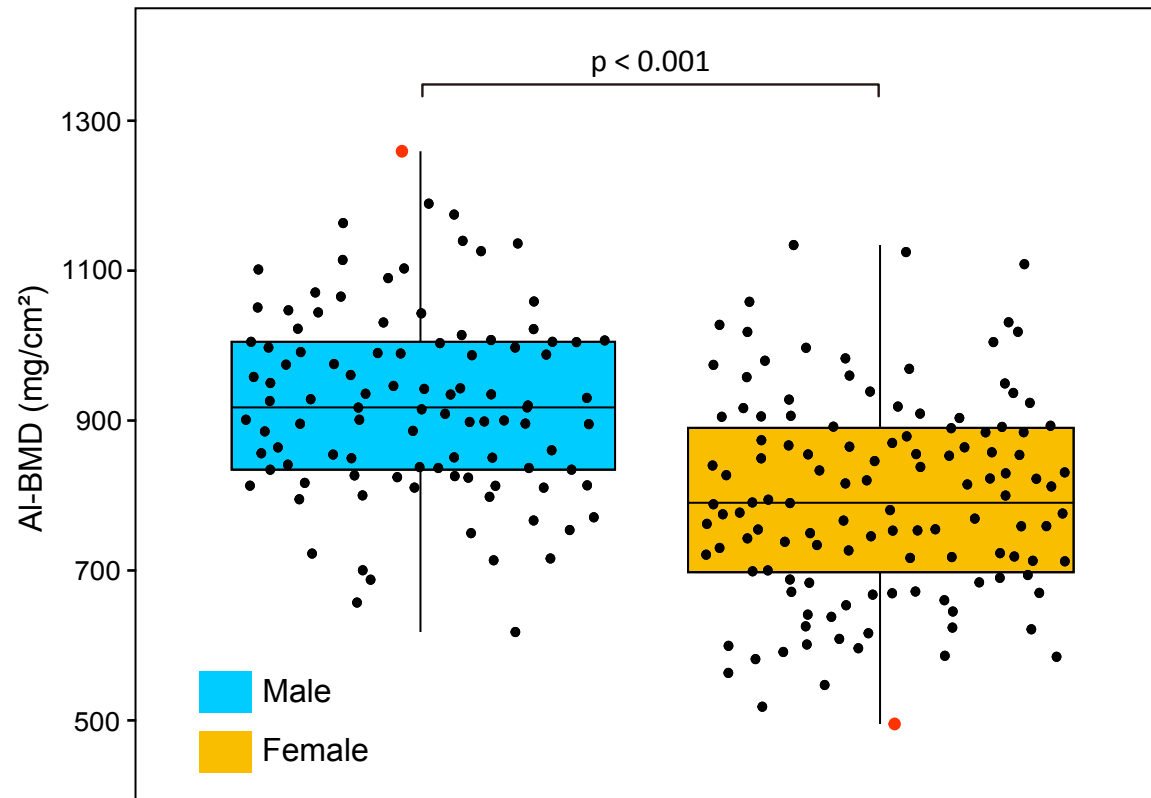

Supplement: Supplementary file 4 — Supplementary Figure S3. [file 41598_2024_57805_MOESM4_ESM.pdf]

## Supplementary Figure S4

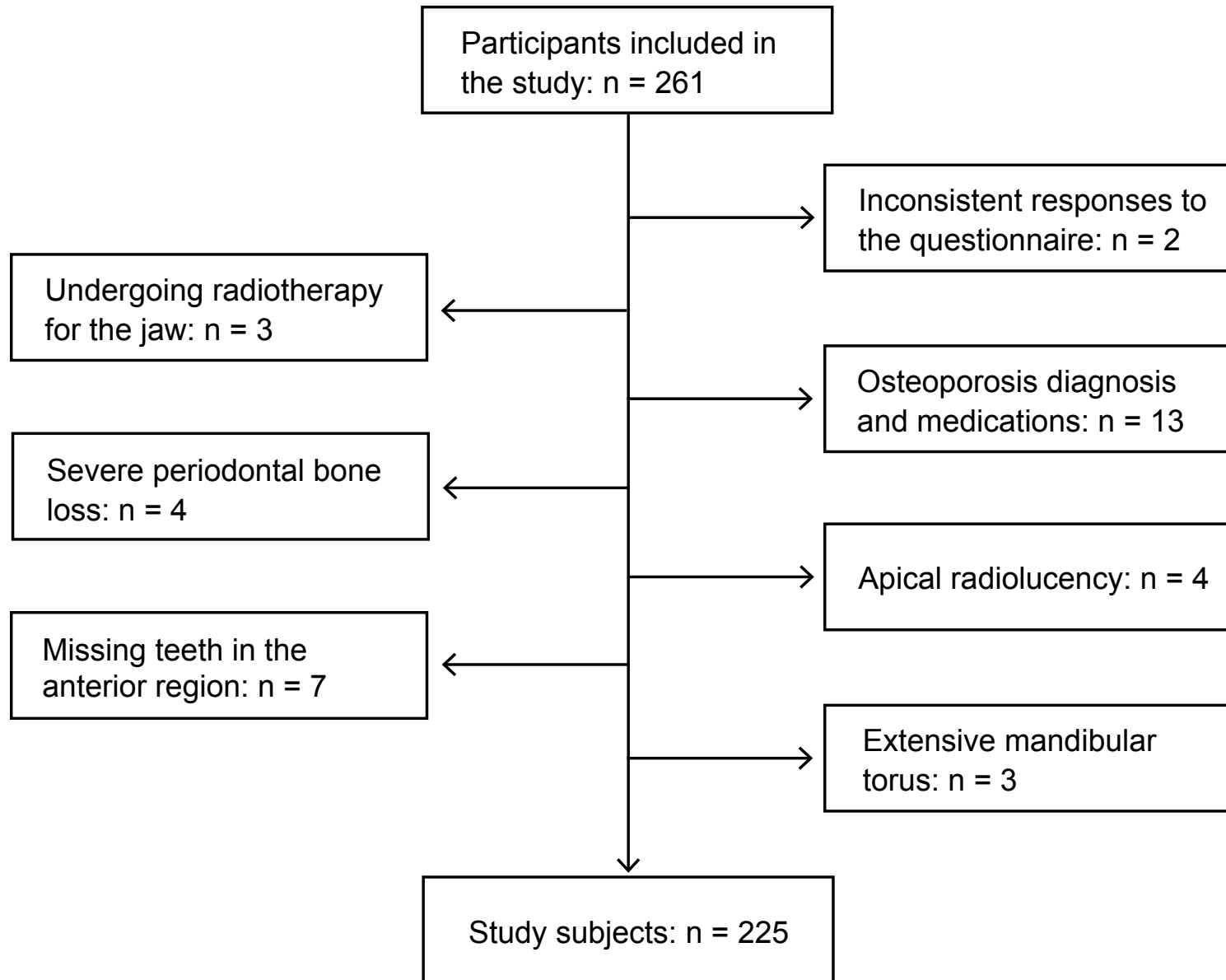

Supplement: Supplementary file 5 — Supplementary Figure S4. [file 41598_2024_57805_MOESM5_ESM.pdf]
