# Supplementary material for: Sex differences and age-related changes in the mandibular alveolar bone mineral density using a computer-aided measurement system for intraoral radiography
Source: Sci Rep. 2024 Mar 28;14:7386. doi: 10.1038/s41598-024-57805-5 (PMC10979020; doi:10.1038/s41598-024-57805-5)
Supplement: Supplementary file 6 — Supplementary Table S1. [file 41598_2024_57805_MOESM6_ESM.docx]

**Supplementary Table S1. Demographic characteristics of the study population**

| Age categories^1)^ | Male |  | Female |  | P-value |
| --- | --- | --- | --- | --- | --- |
|  | N (%) | Mean ± S.D. | N (%) | Mean ± S.D. |  |
| Total | 101 (100%) | - | 124 (100%) | - | - |
| 25–49 years | 22 (21.8%) | 39.6 ± 8.1 | 33 (26.6%) | 39.5 ± 6.7 | 0.809 |
| 50–74 years | 54 (53.5%) | 65.9 ± 7.1 | 69 (55.7%) | 63.4 ± 7.5 | 0.063 |
| >75 years | 25 (24.8%) | 78.0 ± 3.0 | 22 (17.7%) | 80.0 ± 4.1 | 0.076 |

^1)^ Nonparametric Mann–Whitney U-test. S.D., standard deviation
